# Plasma exchange combined with eculizumab in the management of atypical hemolytic uremic in pediatric patients: A case report

**DOI:** 10.1097/MD.0000000000042090

**Published:** 2025-04-11

**Authors:** Lian Yang, Fan Liu, Xiaolu Li, Lanfen He, Yan Gu, Mingcan Sun, Zhenzhen Liu, Zeng Liu

**Affiliations:** a Department of Nephrology, Wuhan Children’s Hospital, Tongji Medical College, Huazhong University of Science and Technology, Wuhan, China; b Wuhan Children’s Hospital West District Comprehensive Service Center, Tongji Medical College, Huazhong University of Science and Technology, Wuhan, Hubei, China.

**Keywords:** atypical hemolytic uremic syndrome, eculizumab, nursing, plasma exchange

## Abstract

**Rationale:**

Atypical hemolytic uremic syndrome (aHUS) is a severe rare disease characterized by microvascular hemolytic anemia, thrombocytopenia, and acute renal failure.

**Patient concerns:**

A 10-year-old male presented with symptoms of weakness, jaundice, pallor, swollen eyelids, and no significant lower limb swelling. Laboratory investigations revealed critical hemoglobin levels, fragmented red blood cells, thrombocytopenia, and acute kidney injury.

**Diagnoses:**

The patient was diagnosed with aHUS based on the clinical trial of microvascular hemolytic anemia, thrombocytopenia, and organ damage due to thrombosis, as well as laboratory findings.

**Interventions:**

The patient was treated with a combination of plasma exchange and the drug Eculizumab. Comprehensive nursing care was provided, including the establishment and maintenance of blood purification pipelines, management of complications (such as coagulation, allergic reactions, hypotension, and bleeding), and psychological support for the patient and family.

**Outcomes:**

The patient successfully completed a 3-month treatment regimen with Eculizumab and has been followed up for an additional 10 months without any recurrence of the condition, demonstrating favorable treatment outcomes.

**Lessons:**

The novel approach of combining plasma exchange with Eculizumab in pediatric aHUS presents significant nursing challenges, but the case demonstrates the potential benefits of this combination therapy, particularly in the pediatric population in China where Eculizumab has recently become available. Comprehensive nursing care, including managing complications and providing psychological support, is crucial to the successful treatment of aHUS.

## 1. Introduction

Atypical hemolytic uremic syndrome (aHUS) is classified among the conditions listed in the First Batch of Rare Diseases Catalogue released by the Chinese government in 2018,^[1]^ with an estimated incidence rate of 5 per 100,000 in children.^[2]^ This syndrome manifests as a triad of microvascular hemolytic anemia, consumption thrombocytopenia, and acute renal failure. Without timely intervention, approximately 25% of affected children succumb to the acute phase, while around 50% progress to end-stage renal disease (ESRD).^[3]^ Anti-factor H anti-body associated aHUS is a unique subgroup of aHUS occurring at any age, but it is more prevalent in the pediatric population.^[4]^ Notably, plasma exchange has demonstrated efficacy in cases of aHUS related to anti-factor H antibodies. In situations where Eculizumab is unavailable, plasma exchange stands as the first-line treatment option and should ideally commence within 24 hours of diagnosis.^[5]^ Eculizumab, a recombinant human anti complement C5 monoclonal antibody, is able to block C5 lysis, inhibit complement terminal activation, reduce endothelial damage and inflammatory response. It has been utilized since 2009 abroad for treating aHUS, showing significant efficacy in patients in the United States and the European Union. Eculizumab was well-established as the first-line treatment for children clinical clinically diagnosed with aHUS.^[6–8]^ In December 2022, Eculizumab became available in China, marking its entry into clinical practice.^[9]^ Despite its potential benefits, there remains a paucity of reports detailing the use of Eculizumab for pediatric aHUS in China. In March 2023, our department admitted a child diagnosed with atypical hemolytic uremic syndrome. Following the diagnosis, plasma exchange was performed to mitigate the progression of the acute phase by removing pathogenic autoantibodies and overactivated complement components due to disagreement of using Eculizumab by the parents. The pediatric patient received Eculizumab after 3 sessions of plasma exchange with agreement of the parents. However, it is essential to acknowledge the Eculizumab falls under the category of clinical new drugs, and the drug instructions list adverse reactions affecting up to 22 types of organs. Commonly reported adverse reactions include headaches, while the most severe adverse reactions include meningococcal sepsis. Moreover, there is a risk of infusion reactions stemming from allergic or hypersensitive reactions to administration. Presently, there is a lack of reports on adverse reactions of Eculizumab’ clinical use in China, increasing the uncertainty and risk associated with infusion treatment. Nevertheless, with timely treatment and meticulouscare, the patient exhibited a favorable prognosis. The following sections detailed the treatment, care, and subsequent follow-up results.

## 2. Case report

### 2.1. Ethical issue

The study was approved by the Ethics Committee of Wuhan Children’s Hospital (no. 2023D019-F01) and written informed consent was collected from the guardian of patient before the study. The study was performed in accordance with the Helsinki II declaration. Written informed consent was obtained from the patient for this case report.

### 2.2. Diagnostic criteria

The diagnostic criteria for aHUS include the manifestation of the clinical triad: microvascular hemolytic anemia, thrombocytopenia, and organ damage due to thrombosis. Additionally, laboratory findings supporting the diagnosis include hemoglobin levels < 100 g/L, presence of fragmented red blood cells on peripheral blood smear, elevated reticulocyte percentage, elevated lactate dehydrogenase levels, and platelet counts <150 × 10^9^/L. Concurrently, acute organ damage, particularly kidney damage, is evident when blood creatinine levels exceed the upper limit of healthy children of the same age and gender.^[5]^

### 2.3. Clinical data

#### 2.3.1. General information

In March 2023, our department admitted a 10-year-old male weighing 27 kg, presenting with weakness, jaundice, pallor, swollen eyelids, and no significant lower limb swelling. Laboratory investigations revealed a hemoglobin concentration of 63 g/L, fragmented red blood cells on peripheral blood smear, a reticulocyte percentage of 6.39%, lactate dehydrogenase levels of 733 U/L, platelet count of 117 × 10^9^/L, creatinine levels of 127 μmol/L, D dimer of 0.81 mg/L, and a serum immune complete set (5 items IgG, IgA, IgM, C3, C4) measurement indicating immunoglobulin A levels of 1.91 g/L and complement C3 levels of 0.58 g/L. In addition, ADAMTS13 was negative. The diagnoses of disseminated intravascular coagulation and thrombotic thrombocytopenic purpura were excluded.

#### 2.3.2. Main treatment and outcomes

**Figure 1.**
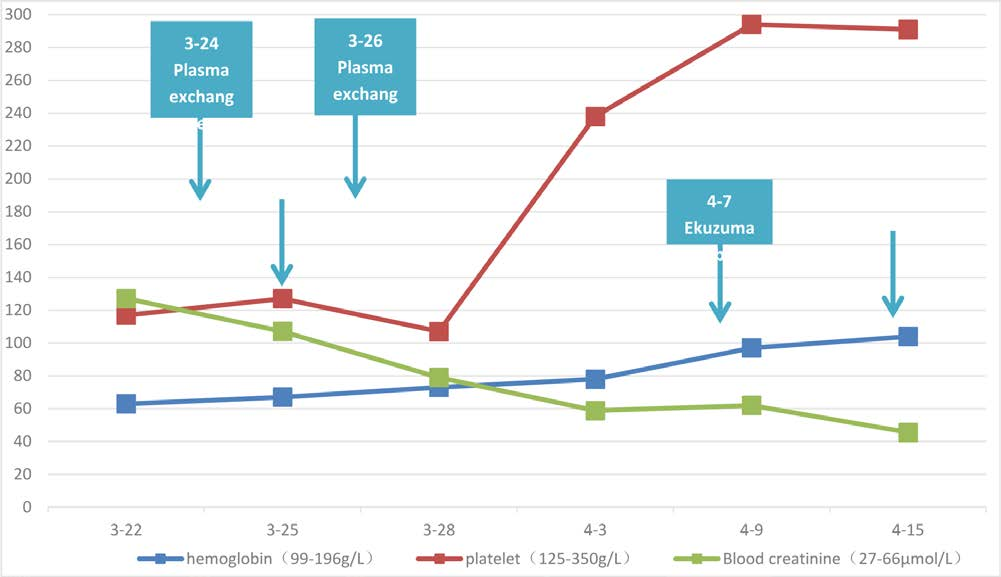
The trend of triad after treatment.

**Figure 2.**
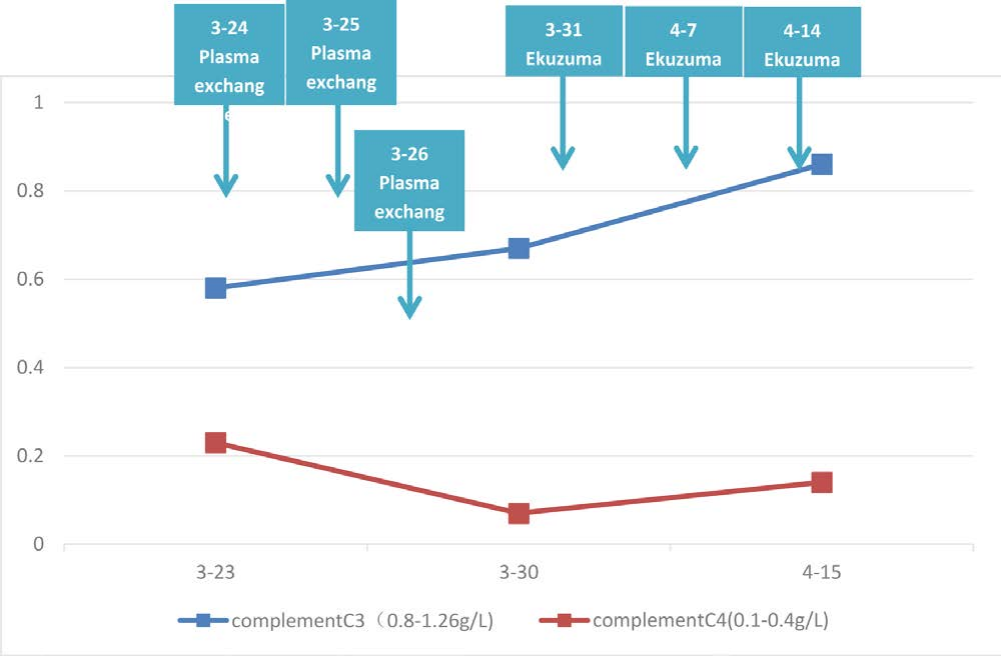
The trend of addiment after treatment.

Upon admission, comprehensive examinations were conducted. Given the critical hemoglobin level of 63 g/L, intravenous administration o fwashed red blood cells at 1 IU was initiated, and the transfusion proceeded smoothly without any adverse reactions. Before the diagnosis of aHUS, 3 cycles of plasma exchange therapy were administered. During the initial session of plasma exchange, the patient developed a rash resembling erythema on the face. Intravenous administration of hydrocortisone succinate (manufactured by Shandong Yantai Dongcheng North Pharmaceutical Co., Ltd., Yantai City, Shandong Province, China, specification: 0.1g/bottle, batch number: 202209161) alleviated the facialrash. Plasma exchange therapy was continued until the completion of treatment, with subsequent sessions progressing smoothly. Following the diagnosis of aHUS, proactive measures were taken to facilitate the procurement of Eculizumab (manufacturer: Alexion Athlete Manufacturing Facility, Athlone, Roscommon, Ireland, specification: 300 mg/bottle, batch number: 1002642), which was administered in 3 doses. Subsequent reevaluation indicated normalization of the “triad” and complement levels, accompanied by the resolution symptoms such as fatigue, eyelid edema, and anemia before discharge. Regular outpatient follow-ups were conducted, with Eculizumab administered 3 times every 2 weeks. Three months after admission, hemoglobin of the pediatric patient recovered to 104 g/L, platelets improved from 117 g/L to 291 g/L, serum creatinine declined from 127.1 μmol/L to 45.5 μmol/L. In addition, GFR was improved from 49.71 ml/min·1.73m^2^ to 138.86 mL/min·1.73m^2^, complement C3 changed from 0.58 g/L to 0.86 g/L, and complement C4 changed from 0.23 g/L to 0.14 g/L. However, proteinuria fluctuated. Over a 10-month follow-up period, all re-evaluation results remained stable within the normal range (Figs. 1 and 2).

### 2.4. Nursing

#### 2.4.1. Observation and nursing of complications in blood purification treatment

##### 2.4.1.1. Coagulation during extracorporeal circulation

Before each plasma exchange treatment, the tubing filter was heparinized by adding 10,000 U of heparin to 2000 mL of pre-filled saline tubing, followed by a 30-minute filtration process.^[10]^ Subsequently, the tubing and filter were rinsed with 1000 mL of saline to prevent the introduction of heparin saline into the patient’s bloodstream, minimizing the risk of bleeding. Anticoagulation was maintained with low molecular weight heparin calcium at a dosage of 60 to 80 IU/Kg, while vigilant monitoring for early signs of extracorporeal circulation coagulation, such as darkened blood color in tubing and filters, hollow fiber coagulation in filters, and small clots in arterial or venous ampulla, was conducted.^[11]^ Fortunately, the patient did not experience any coagulation during extracorporeal circulation throughout the blood purification treatment.

##### 2.4.1.2. Allergies and allergic reactions

To preemptively mitigate allergic reactions, the patient received oral loratadine syrup, dexamethasone, and intravenous calcium gluconate before each plasma exchange. Despite encountering a facial rash during the first plasma exchange, accompanied by itching and chills, the situation was promptly managed with the administration of 100 mg intravenous hydrocortisone sodium succinate. Subsequent plasma exchange treatments proceeded without incident of allergic reactions.

##### 2.4.1.3. Hypotension

Hypotension represents the most common complication of blood purification therapy.^[12]^ According to the patient’s surface area, the Asahi Chemical OP-05W membrane-type plasma separator^[13]^ and the disposable blood circuit pipeline for pediatric artificial kidney (168 mL) were selected. Before initiating treatment, careful observation of the patient’s vital signs such as blood pressure, heart rate, and respiration was conducted. Throughout the treatment process, continuous monitoring of vital signs and blood oxygen saturation was maintained. In case of a decrease in blood pressure, plasma output was slowed down, and the plasma exchange duration was extended. In severe cases, vasoactive drugs were administered or the treatment was discontinued.^[14]^ Fortunately, the child did not experience hypotension.

##### 2.4.1.4. Hypocalcemia

Hypocalcemia is a potential complication of plasma replacement therapy.^[13]^ To mitigate the risk of hypocalcemia, low molecular weight heparin calcium was administered for anticoagulation, and 10 ml of 10% calcium gluconate was slowly injected intravenously before and during plasma support, as per the physician’s instructions. Closely monitoring for symptoms such as limb numbness and muscle twitching was carried out during treatment, fortunately, without any incidence of hypocalcemia.

##### 2.4.1.5. Bleeding

Platelet destruction during the replacement process, combined with anticoagulant use and the inherent low platelet levels of the disease, increases the risk of bleeding. Prior to treatment, thorough inspection for skin or mucosal ecchymosis and absence of blood leakage or hematoma at the puncture site was performed. During the operation, an appropriate dosage of low molecular weight heparin calcium anticoagulation was administered based on the child’s coagulation function and body mass. Gentle operation techniques were employed, with close monitoring of the patient’s skin, mucosa, and catheterization site for signs of bleeding. Fortunately, no bleeding occurred during treatment.

#### 2.4.2. Observation and nursing of adverse reactions to eculizumab

##### 2.4.2.1. Prevention of antibiotic use

Hai et al conducted a review of 20 Chinese and English literature sources on Eculizumab, encompassing 131 cases, revealing that 81.7% of patients developed Neisseria meningitidis infection post-Eculizumab administration, aligning with the adverse reaction profile outlined in the drug instructions. Despite adherence to regulations mandating vaccination against Group A and Group C meningococcal polysaccharide at least 2 weeks prior to Eculizumab treatment, breakthrough infections persist in most cases. Consequently, Europe, the United States, and France advocate for antibiotic prophylactic treatment. As a precautionary measure, the patient opted for oral prophylactic azithromycin, effectively averting symptoms of meningitis during the treatment period.

##### 2.4.2.2. Drugs for preventing adverse reactions

Literature reports indicate 91% of patients encounter noninfectious adverse reactions, with intravenous allergy reactions being predominant within 1 month post-Eculizumab.^[15]^ To preemptively mitigate potential allergic reactions, 5 mL of loratadine syrup was orally administered prior to Eculizumab treatment. Fortunately, the patient remained free from allergic reactions throughout the Eculizumab treatment.

##### 2.4.2.3. Treatment with eculizumab

The recommended dosage and dosing interval for Eculizumab, as per the latest guidelines, are detailed in Table 1.^[5]^ Tailored to the patient’s weight, a dose of 600 mg/dose of Eculizumab was selected. Following medical guidance, 60 mL of Eculizumab was mixed with 60 mL of 5% glucose injection to achieve a concentration of 5 mg/mL. Initiation of infusion via an infusion pump commenced at a rate of 25 mL/h. Throughout the infusion process, meticulous monitoring of the patient’s vital signs was conducted, alongside vigilant observation for any discomfort, such as skin itching, cough, joint pain, or fever, every 15 to 30 minutes. Remarkably, the patient remained devoid of discomfort symptoms, prompting a gradual adjustment of the pump speed to 50 mL/h. Infusion was meticulously completed within 1 to 4 hours, adhering strictly to pediatric patient instructions. Pre- and post-infusion, 5% glucose injection was administered through a flushing tube. Encouragingly, the patient experienced no discomfort symptoms, such as skin itching, cough, or joint pain, throughout the treatment period.

**Table 1:** Dosage and dosing interval of ekuzumab.

Weight	Load dosage	Maintenance dosage
≥40 kg	900 mg/week × 4 weeks	1200 mg the 5th week, 1200 mg followed every 2 weeks
30–40 kg	600 mg/week × 2 weeks	900 mg the 3rd week, 900 mg followed every 2 weeks
20–30 kg	600 mg/week × 2 weeks	600 mg the 3rd week, 600 mg followed every 2 weeks
10–20 kg	600 mg/week × 1 weeks	300 mg the 2nd week, 300 mg followed every 2 weeks
5–10 kg	300 mg/week × 1 weeks	300 mg the 2nd week, 300 mg followed every 3 weeks

## 3. Discussion

aHUS represents a critical thrombotic microvascular disease and stands as a primary cause of acute kidney injury in children. The common characteristics to all forms of aHUS is the presence of endothelial cell lesions in the microvasculature of the kidney and, less frequently, of other organs. There is no strong evidence for plasma therapy efficacy in aHUS. However, plasma exchange is endorsed as a first-line treatment in the absence of Eculizumab availability in China,^[16]^ while its standalone recurrence rate is alarmingly high, reaching 40%. Instances of multiple relapses post-plasma exchange and corticosteroid shock therapy have been reported, accompanied by rapid renal function decline. The 2016 management plan for pediatric aHUS advocated for Eculizumab as the preferred first-line treatment.^[6]^ In the absence of ADAMTST13 results, thrombotic thrombocytopenic purpura cannot be ruled out. Hence, prompt initiation of plasma exchange therapy is imperative, with Eculizumab emerging as the preferred clinical intervention upon a diagnosis of aHUS. Eculizumab tolerability is generally good. Eculizumab for treating aHUS has been tested in previous studies. Eculizumab maintained haematological normalization in 90% of patients despite plasma therapy cessation, and was associated with a small but significant increase in estimated GFR.^[17,18]^ The increase in GFR was more pronounced in children^[19,20]^ than in adults.^[17,18]^ Indeed, the time between onset of aHUS episode and treatment initiation inversely correlates with the increase in GFR.

The patient’s severe condition and challenges in blood purification treatment underscore the complexity of managing aHUS in children. The novel approach of combining plasma exchange with Eculizumab in pediatric aHUS, unreported domestically, presents significant nursing challenges. General nurses underwent training on Eculizumab-related knowledge, and a designated dialysis nurse was assigned to oversee plasma exchange and Eculizumab treatment, ensuring uninterrupted blood dialysis pipeline maintenance and vigilant monitoring of condition and vital signs. Emphasis was placed on managing plasma exchange complications and adverse drug reactions through preventive nursing measures, alongside prioritizing patient emotional comfort and family psychological support to enhance treatment compliance. The patient has been followed up for 10 months, adhering to the Eculizumab treatment regimen, with no recurrence and positive treatment outcomes.

While the combination of plasma exchange and Eculizumab yields improved therapeutic outcomes in aHUS, lifelong administration of either treatment is not recommended. Further research is warranted to delineate the optimal treatment plan and cycle for Eculizumab in aHUS management in China. Presently, the patient in this report as successfully completed a 3-month treatment regimen with Eculizumab as planned and has been followed up for an additional 10 months without any recurrence of the condition, demonstrating favorable treatment outcomes. Furthermore, with the inclusion of Eculizumab in the national medical insurance drug catalog, the cost of the medication has significantly decreased by nearly 90%. This substantial reduction in drug expenses has bolstered the confidence of patients’ families in actively opting for Eculizumab treatment. Previous studies reported that none of the children and only 1 (1%) of the 78 adults died within the 1 to 2 years of study follow-up with use of eculizumab.^[17,18,21]^ As of the completion of this study, our department administered Eculizumab treatment to a total of 5 patients diagnosed with aHUS. All patients received a 3-month Eculizumab treatment plan, and all achieved satisfactory treatment outcomes. Currently, all 5 patients have undergone treatment or follow-up examinations as per the established protocol. However, a longer follow-up duration is needed to ascertain whether continued administration of Eculizumab is necessary.

## Acknowledgments

We appreciate the patient for participating in this study.

## Author contributions

**Conceptualization:** Lian Yang, Fan Liu, Xiaolu Li, Zeng Liu.

**Data curation:** Lian Yang, Fan Liu, Lanfen He, Yan Gu, Zhenzhen Liu, Xiaolu Li, Zeng Liu.

**Formal analysis:** Lian Yang, Fan Liu, Lanfen He, Yan Gu, Mingcan Sun, Zhenzhen Liu.

**Investigation:** Lanfen He, Mingcan Sun, Xiaolu Li, Zeng Liu.

**Methodology:** Yan Gu, Mingcan Sun.

**Software:** Xiaolu Li.

**Writing – original draft:** Lian Yang, Zeng Liu.

**Writing – review & editing:** Zhenzhen Liu, Xiaolu Li, Zeng Liu.
